# Stably transmitted defined microbial community in honeybees preserves *Hafnia alvei* inhibition by regulating the immune system

**DOI:** 10.3389/fmicb.2022.1074153

**Published:** 2022-12-01

**Authors:** Jieni Wang, Haoyu Lang, Wenhao Zhang, Yifan Zhai, Li Zheng, Hao Chen, Yan Liu, Hao Zheng

**Affiliations:** ^1^College of Food Science and Nutritional Engineering, China Agricultural University, Beijing, China; ^2^Faculty of Agriculture and Food, Kunming University of Science and Technology, Kunming, China; ^3^Shandong Academy of Agricultural Sciences, Institute of Plant Protection, Jinan, China; ^4^Key Laboratory of Natural Enemies Insects, Ministry of Agriculture and Rural Affairs, Jinan, China

**Keywords:** *Apis mellifera*, colonization resistance, *Hafnia alvei*, immune system, gut microbiota

## Abstract

The gut microbiota of honeybees is highly diverse at the strain level and essential to the proper function and development of the host. Interactions between the host and its gut microbiota, such as specific microbes regulating the innate immune system, protect the host against pathogen infections. However, little is known about the capacity of these strains deposited in one colony to inhibit pathogens. In this study, we assembled a defined microbial community based on phylogeny analysis, the ‘Core-20’ community, consisting of 20 strains isolated from the honeybee intestine. The Core-20 community could trigger the upregulation of immune gene expressions and reduce *Hafnia alvei* prevalence, indicating immune priming underlies the microbial protective effect. Functions related to carbohydrate utilization and the phosphoenolpyruvate-dependent sugar phosphotransferase system (PTS systems) are represented in genomic analysis of the defined community, which might be involved in manipulating immune responses. Additionally, we found that the defined Core-20 community is able to colonize the honeybee gut stably through passages. In conclusion, our findings highlight that the synthetic gut microbiota could offer protection by regulating the host immune system, suggesting that the strain collection can yield insights into host-microbiota interactions and provide solutions to protect honeybees from pathogen infections.

## Introduction

The host intestinal tract is a complex ecosystem offering niches for beneficial symbionts that aid in food digestion and disease resistance (Engel and Moran, 2013b; Pereira and Berry, 2017). Imbalanced gut microbiota driven by the antibiotic treatment could lead to metabolism changes, potentially pathogenic bacteria blooming, epithelial barrier disruption, and increased susceptibility to infections (Buffie et al., 2015; Raymann et al., 2017; Fünfhaus et al., 2018; Lang et al., 2022). Therefore, the gut microbiota can preclude infections of enteric pathogens, which is one of the most widespread benefits to its host (Spees et al., 2013; Kim et al., 2017). Considering the complexity of interactions between the microbiota and the host, the underlying basis of this protection, or ‘colonization resistance’, is still insufficiently understood.

Honeybees (*Apis mellifera*) harbor about five core host-specific bacterial genera, which probably have co-evolved with social bees for over 80 million years (Koch et al., 2013; Kwong and Moran, 2016). They include *Snodgrassella*, *Gilliamella*, *Bifidobacterium*, *Bombilactobacillus* Firm-4, and *Lactobacillus* Firm-5 (Martinson et al., 2011; Kwong and Moran, 2016). Additionally, the genus *Apilactobacillus*, *Frischella*, *Commensalibacter, Bartonella*, and *Bombella* are less prevalent, which occupy particular niches and engage in host health maintenance (Engel et al., 2016; Liu et al., 2022). With relatively simple gut microbiota, honeybees present opportunities to investigate gut community dynamics and host–microbe interaction as an experimental system (Zheng et al., 2018). Recent research has demonstrated the honeybee gut microbiome contributes to metabolism, development, and protection against pathogens (Engel et al., 2016; Raymann and Moran, 2018). Some species belonging to *Bombilactobacillus* Firm-4, *Lactobacillus* Firm-5, and *Bifidobacterium* can inhibit the growth of other microorganisms *in vitro* (Forsgren et al., 2010; Vásquez et al., 2012; Butler et al., 2013; Killer et al., 2014). Members of bee gut microbiota, such as *Snodgrassella alvi* and *Gilliamella apis*, could lower gut lumen pH and oxygen levels (Zheng et al., 2017), compete for nutrients (Martinson et al., 2012; Wu et al., 2021), and antagonize with type VI secretion system (Steele et al., 2017) to inhibit pathogen virulence and growth.

The colonization resistance conferred by the gut microbiota through stimulating the host’s innate immune system was supported by increasing evidence (Lawley and Walker, 2013). The innate immune system of honeybees comprises the Toll and Imd pathways (Lourenço et al., 2013, 2018; Danihlík et al., 2015), which primarily regulate the production of antimicrobial peptides (AMPs), such as abaecin, apidaecin, defensin, and hymenoptaecin, during pathogen infection (Evans et al., 2006; Guo et al., 2021). When honeybees were colonized with conventional gut microbiota or mono-colonized with strains from *S. alvi*, the immune system of honeybees was stimulated to inhibit potential pathogens such as *Serratia marcescens* (Horak et al., 2020). However, substantial strain-level diversity was found within the bee gut microbiota, where individual strains harbor unique genes and distinct functional capabilities (Ellegaard et al., 2019; Brochet et al., 2021; Lang et al., 2022). In addition to understanding individual strains involved in interactions determining colonization resistance, how bacterial combinations by multiple strains from different species control colonization resistance still need to be investigated.

*Hafnia alvei*, a specific pathogen in bees, could cause septicemia with a mortality rate of over 90% by injection and inflammation of the intestinal tract by oral (Møller, 1954; Erban et al., 2017; Grabowski and Klein, 2017). Leveraging previous work, *Lactobacillus apis* W8171 could inhibit *H. alvei* infection and prevent severe mucosal architecture damage in the honeybee rectum (Lang et al., 2022). In this study, we established a consortium based on phylogeny analysis, the ‘Core-20’ community, consisting of 20 strains isolated from the honeybee intestine that provide colonization resistance against *H. alvei*. Interestingly, the higher complex and biodiversity community displays advantages in promoting the expression of regulators and AMPs of the immune system. The comparative genomic analysis revealed that the phosphoenolpyruvate-dependent sugar phosphotransferase system (PTS system) could potentially be involved in manipulating immune responses. In addition, we transmitted the Core-20 community for four passages and found that the Core-20 could colonize steadily. Thus, the Core-20 community serves as a stable and functional microbiota that can be used for detailed investigation of host-microbe and microbe-microbe interactions in honeybees.

## Materials and methods

### Characterization of stains in the Core-20 community designed by the phylogeny of honeybee gut microbiota

**Figure 1:**
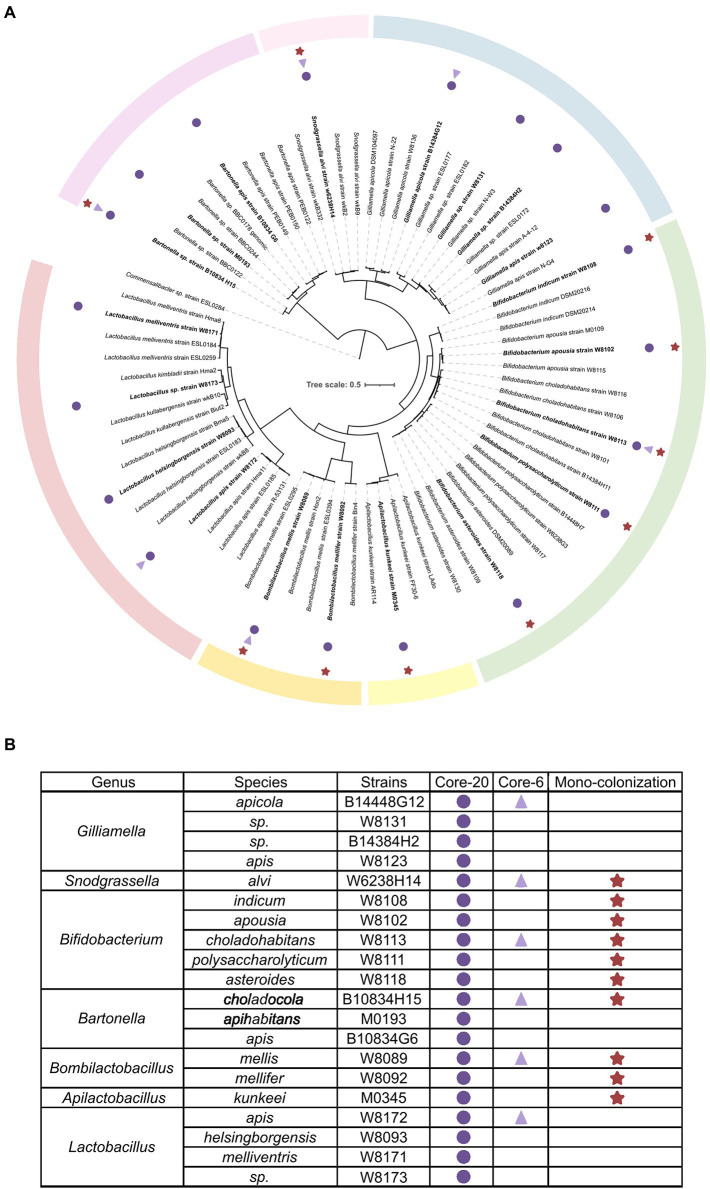
Composition of honeybee gut microbiota and strains of the Core-20 and Core-6 community. **(A)** Maximum-likelihood tree inferred by GTDB-tk based on the amino acid sequences of bacterial marker genes. **(B)** Detailed information on strain classification and grouping. The Core-6 community consists of six strains representing the six most prevalent and abundant genera of honeybee gut microbiota, and the Core-20 is composed of 20 strains at the species level. Rounds mark the strains of the Core-20, triangles mark the members of the Core-6 and stars mark strains used in the mono-colonization experiments. Color bars indicate the classification of honeybee gut microbiota.

To establish defined minimal microbiota that recapitulates healthy honeybee gut microbiota stably and functionally, the integral intestine homogenization of conventional honeybee was cultured on a rich, non-selective culture medium. About 110 strains were mono-cloned and identified by whole genome sequencing (WGS), representing conventional bacterial strains. The quality-controlled reads were assembled with the SOAPdenovo2 genome assembler. The completeness and contamination of genomes were assessed by CheckM (>96% completeness, <0.6% contamination). Phylogenetic analysis by WGS shows that strains assorted into different clusters according to gANI identities referred to as species-level (Su et al., 2021; Wu et al., 2021). Six strains representing the six most prevalent and abundant genera of honeybee gut microbiota are selected for a bacterial consortium named “Core-6,” and 20 strains at the species-level form “Core-20” bacterial community (Figure 1).

Within the *Proteobacteria* phylum, four members of the Core-20 community were assigned to the genus *Gilliamella*, one strain to *Snodgrassella,* and three strains to *Bartonella*. Two abundant species clusters in the *Firmicutes* phylum are *Bombilactobacillus* Firm-4 and *Lactobacillus* Firm-5, including two strains and four strains, respectively. Additionally, *Apilactobacillus kunkeei*, which proved its ability to protect honeybees from pathogens, was added as an essential functional part (Daisley et al., 2020a,b).

Bacterial strains were isolated from the guts of *A. mellifera* and stored at –80°C with 25% (v/v) glycerol PBS solution. The glycerol stocks were plated on heart infusion agar supplemented with 5% (vol/vol) defibrinated sheep’s blood (Solarbio, Beijing, China), MRS agar (Solarbio, Beijing, China) or TPY agar (Solarbio, Beijing, China) incubated at 35°C in 5% CO2 for 2–3 days. The culture conditions of strains used in this study were described by Wu et al. (2021). Confirmed by PCR with universal bacterial primers 27F (5′-AGAGTTTGATCCTGGCTCAG-3′) and 1492R (5′-TACGACTTAACCCCAATCGC-3′), individual strains were mixed with 25% glycerol PBS solution. The defined bacterial communities were generated by mixing equal volumes of bacterial suspensions with adjusted OD_600_ = 1.

### Honeybee collection, containment, and experiment

Microbiota-free (MF) bees were obtained as described by Zheng et al. (Zheng et al., 2018). All bees were kept in an incubator (35°C, RH 50%). For the *H. alvei* challenging experiment, newly emerged MF bees (Day 1) were divided into several groups, with 25 MF bees in one cup cage. For each colonization group, bees lived on the 1 ml bacterial suspensions mixed with 1 ml sucrose solution (50%, w/v) and 0.5 g sterilized pollen for 24 h. For the MF group, 1 ml of 1 × PBS was mixed with 1 ml of sucrose solution (50%, w/v) and 0.5 g sterilized pollen. After 24 h inoculation, all groups were fed regular diets, sucrose solution (50%, w/v), and sterilized pollen. To precisely control the infection amount of *H. alvei* cells, bees from the colonization and MF groups were all orally inoculated with *H. alvei* SMH01 individually on Day 7 (Lang et al., 2022). After five-day regular diets, the load of *H. alvei* was determined by qPCR.

For inoculation and sampling in passaging line, newly emerged MF bees (Day 1) were randomly assigned to three cups and living on the 1 ml the Core-20 bacterial suspension mixed with 1 ml sucrose solution (50%, w/v) and 0.5 g sterilized pollen for 24 h, with 25 MF bees in one cup cage. Five days after the final oral inoculation, the whole gut of each bee was sampled, immediately placed into a sterile 1.5 ml tube individually, and ground with sterile 25% glycerol PBS solution. Three guts from each cup were pulled together to prepare inoculation for the following passage, and the other guts were stored at −80°C for DNA extraction and sequencing.

### 16S rRNA gene amplicon sequencing and processing

DNA was extracted from gut homogenates using the CTAB method (Powell et al., 2014; Zheng et al., 2018). Targeted amplicons of the V3-4 region of the 16S rRNA gene were generated with primers 341F and 806R (Caporaso et al., 2011). Sequencing libraries were generated with NEBNext Ultra II DNA Library Prep Kit for Illumina (New England Biolabs, Ipswich, United States). They were sequenced at Novogene Bioinformatics Technology Co. Ltd., Beijing, China, on the Illumina NovaSeq6000 platform (2 × 250 bp). Bioinformatic analysis was implemented using Mothur (version 1.40.5; Schloss et al., 2009; Kozich et al., 2013; Schloss, 2020). After primer trimming and quality control, sequences were split into groups corresponding to their taxonomy at the level of species and then assigned to operational taxonomic units (OTUs) at a 1% dissimilarity level based on the reference database consisting of aligned 16S rRNA sequences of our 20 strains (Supplementary Figure S1; Xue et al., 2019). Relative abundances were then calculated based on the read numbers. Principal coordinates analysis (PCoA) and alpha diversity indices were visualized in R (version 3.6.1). Raw sequence reads have been deposited at the NCBI SRA database under the BioProject accession number PRJNA891025.

### Quantitative PCR of bacterial 16S rRNA genes and immune-related genes

DNA was extracted from gut homogenates using the CTAB method (Powell et al., 2014; Zheng et al., 2018). DNA concentration was determined with the Qubit 4 Fluorometer (Thermo Fischer Scientific; Waltham, MA, United States). *H. alvei* loads and immune-related gene expressions were determined by qPCR using the ChamQ Universal SYBR qPCR Master Mix (Vazyme Biotech, Nanjing, China). Primer sets specific to *H. alvei* and immune-related genes are listed in Supplementary Table S1 (Horak et al., 2020; Lang et al., 2022). The primers of spaetzle 4 (*Spz4*; XM_028668966.1) were designed based on the nucleotide sequence available in GenBank: forward 5′-CAACGAATTCAGGGACGAGG-3′, reverse 5′-AGTAGTGCCGGGGAAATTCA-3′. All qPCRs were performed in 96-well microplates on a QuantStudio 1 real-time PCR system (Thermo Fischer Scientific). Melting curves were generated after each run (95°C for 15 s, 60°C for 20 s, and increments of 0.3°C until reaching 95°C for 15 s). Each reaction was performed in triplicates on the same plate. The data was analyzed using the QuantStudio Design and Analysis Software. After calculating gene copies, normalization was performed to reduce the effect of gut size variation and extraction efficiency using the host’s actin gene (Kešnerová et al., 2020).

### Functional genomics analysis

Input files were assembled and annotated genomes of the Core-20 (Su et al., 2021; Wu et al., 2021). *H. alvei* reference protein sequence (GCF_011617105.1) was downloaded from NCBI and annotated by KAAS^1^ (Moriya et al., 2007). Artificial metagenomes were created by merging the contigs of each genome into a multi-fasta file (Brugiroux et al., 2016). KEGG mapping was performed using the online version^2^ (Kanehisa et al., 2022). The comparison and analysis of orthologous clusters among genomes were performed at^3^ (Xu et al., 2019).

### Statistical analysis

Statistical analysis was performed using one-way ANOVA (ANalysis Of VAriance) with post-hoc Tukey HSD (honestly significant difference) using package “multcomp” in R (version 3.6.1). *p*-value of less than 0.05 (two-tailed) was considered statistically significant (*p < 0.05, **p < 0.01, ***p < 0.001).

## Results

### Resistance of the Core-20 community against honeybee opportunistic pathogen *Hafnia alvei*

**Figure 2:**
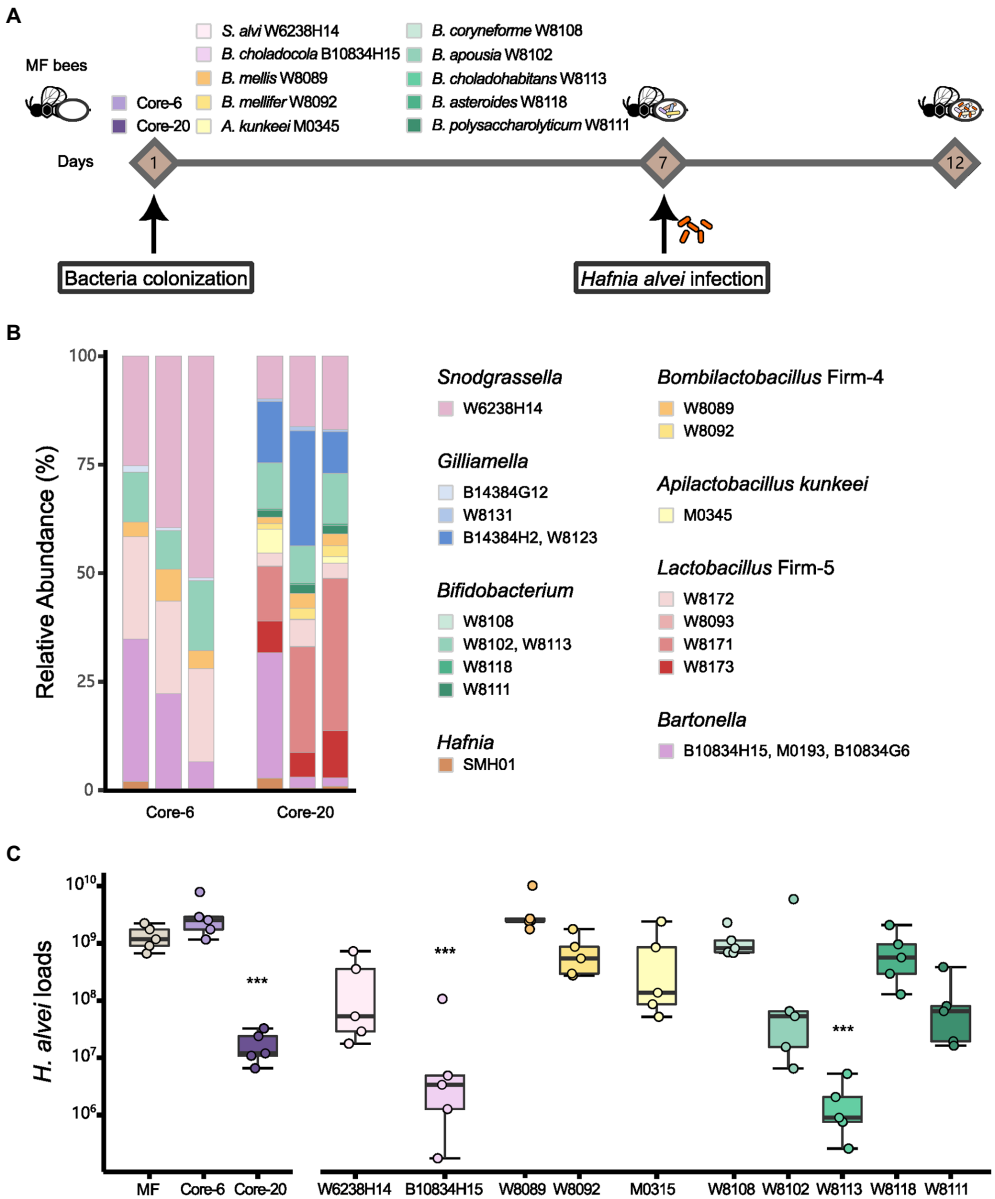
Core-20 leads to protection against oral *H. alvei* infection. **(A)** Experimental design for honeybees colonized with specific microbes challenging with *H. alvei*. **(B)** Relative abundance of the Core-6 and Core-20 community on Day 12. 16S rRNA V3-V4 amplicons were sequenced and analyzed, showing successful microbiota colonization and composition differences between the Core-6 and the Core-20. **(C)** Absolute abundance of *H. alvei* in different treatment groups 5 days post-infection. Single strains, such as *B. choladocola* B10834H15 and *B. choladohabitans* W8113, significantly inhibited the growth of *H. alvei*. The Core-20 community, which is much more complex than the Core-6, significantly reduced the *H. alvei* loads.

To evaluate the potential of the defined communities to protect against *H. alvei* infection, we first colonized MF honeybees with the Core-20, the Core-6, and strains from the genus *Snodgrassella*, *Bartonella*, *Bombilactobacillus* Firm-4, *Apilactobacillus* and *Bifidobacterium* (Figure 1). At Day 7, all honeybee were orally infected with *H. alvei* individually (10^6^ CFU per bee; Figure 2A). Successful microbiota colonization was confirmed by 16S rRNA gene V3-V4 amplicon sequencing at Day 12. Compositional analysis showed that observed species in the Core-20, compared to the Core-6, were increased, and the relative abundance of each taxonomy group differs (Figure 2B). Strains W8131, B14384H2 and W8123 from the genus *Gilliamella* and strains W8093, W8171, and W8173 from the genus *Lactobacillus* Firm-5, which are specific to the Core-20, exhibit substantial improvement in species abundances, showing their fitness in honeybee gut environment and ability to coexist with the complex bacterial community.

After 5 days of infection, *H. alvei* loads were measured by qPCR. Among mono-colonized bees, only *B. choladocola* B10834H15 and *B. choladohabitans* W8113 significantly inhibited the growth of *H. alvei in vivo* compared with the MF group at Day 12 (Figure 2C). According to our previous research, *H. alvei* loads in the bees with *L. apis* W8172, and *Gilliamella apicola* W8136 (the same species as *G. apicola* B14384G12) were also significantly lower than MF bees (Lang et al., 2022). Interestingly, bees treated with the Core-6, including all these strains demonstrating the ability to inhibit pathogens, did not show a significant reduction of *H. alvei*, while the Core-20 reduced the *H. alvei* loads by 78 times. Taken together, the presence of particular species did inhibit *H. alvei*. Still, this microbiota-induced prevention of pathogen infection possibly changes with the gut microbiota composition, suggesting a complex dynamic balance between microbe-host and microbe-microbe interaction.

### Immune expression response induced by the defined community

**Figure 3:**
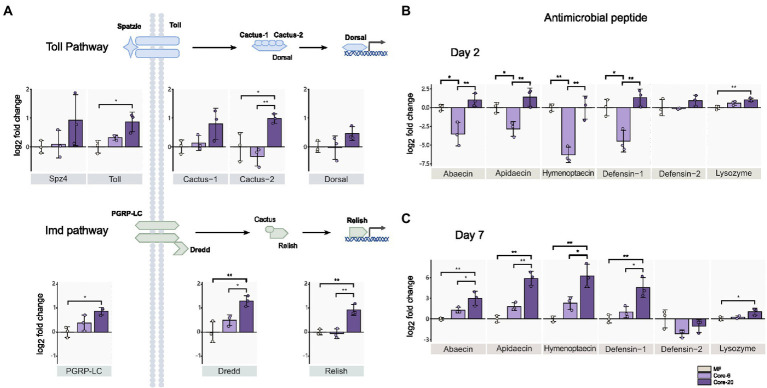
Core-20 triggers host immune gene expression in Imd and Toll pathways **(A,B)** at 24 h post-colonization and **(C)** 7 days post-colonization. The Core-20 displayed a significant promotion in regulators of the Toll and Imd pathways on Day 2 and potential ongoing upregulation in AMPs expression on Day 7. Besides, the Core-6 significantly reduced the expression of AMPs on Day 2. All results indicated that the gut microbiota could stimulate the host’s innate immune system. Gene expression was normalized relative to the housekeeping gene *actin*. *p < 0.05; **p < 0.01 (Tukey honest method).

Intestinal homeostasis maintenance depends on dynamic interactions between gut bacteria and the host’s innate immune systems (Yoo et al., 2020). Commensal gut microbiota could prevent pathogen colonization and infection by enhancing the mucosal barrier and promoting innate immune responses. The gut microbial symbionts of the honeybee can induce antimicrobial immune responses in the host, like AMPs (Kwong et al., 2017). We assessed the relative expression of genes from Toll and Imd pathways by qPCR 24 h following inoculation with the Core-20 and Core-6. The Toll and Imd pathways include the receptors (spz4, toll; pgrp-lc), the regulators (cactus; dredd), and the transcription factors (dorsal; relish), respectively. On Day 2, bees colonized with the Core-20 significantly upregulated *pgrp-lc*, *dredd,* and *relish* from the Imd pathway as well as *toll* and *cactus-2* from the Toll pathway relative to MF bees (Figure 3A). Furthermore, we focused on the expression of genes encoding AMPs, and remarkably, we discovered that bees with the Core-6 exhibited a notable reduction of AMPs *abaecin*, *apidaecin*, *hymenoptaecin* and *defensin-1* (Figure 3B), which might indicate an immunosuppressive ability of the Core-6. To find out whether the Core-20 could consistently upregulate host-producing AMPs in response to *H. alvei* infection, the expression of AMPs genes was measured again on Day 7, right before *H. alvei* inoculation. Interestingly, bees with the Core-20 showed a significant increase of AMPs *abaecin*, *apidaecin*, *hymenoptaecin,* and *defensin-1* (Figure 3C), indicating abilities of the Core-20 to stimulate innate immune response preventing the colonization by pathogenic *H. alvei*.

Our findings showed the Core-6 community exhibited diminished production of antimicrobial peptides, while the Core-20 community upregulated the host immune system, including regulators in innate immune pathways and AMPs expression. *Apidaecin*, the most susceptible AMP against *H. alvei in vitro* (Lang et al., 2022), expressed much higher in the Core-20 condition. Overall, our findings suggested that a primary mechanism by which Core-20 provides colonization resistance is that it can trigger host immune responses.

### Potential to regulate host immune system through phosphoenolpyruvate-dependent sugar phosphotransferase system

**Figure 4:**
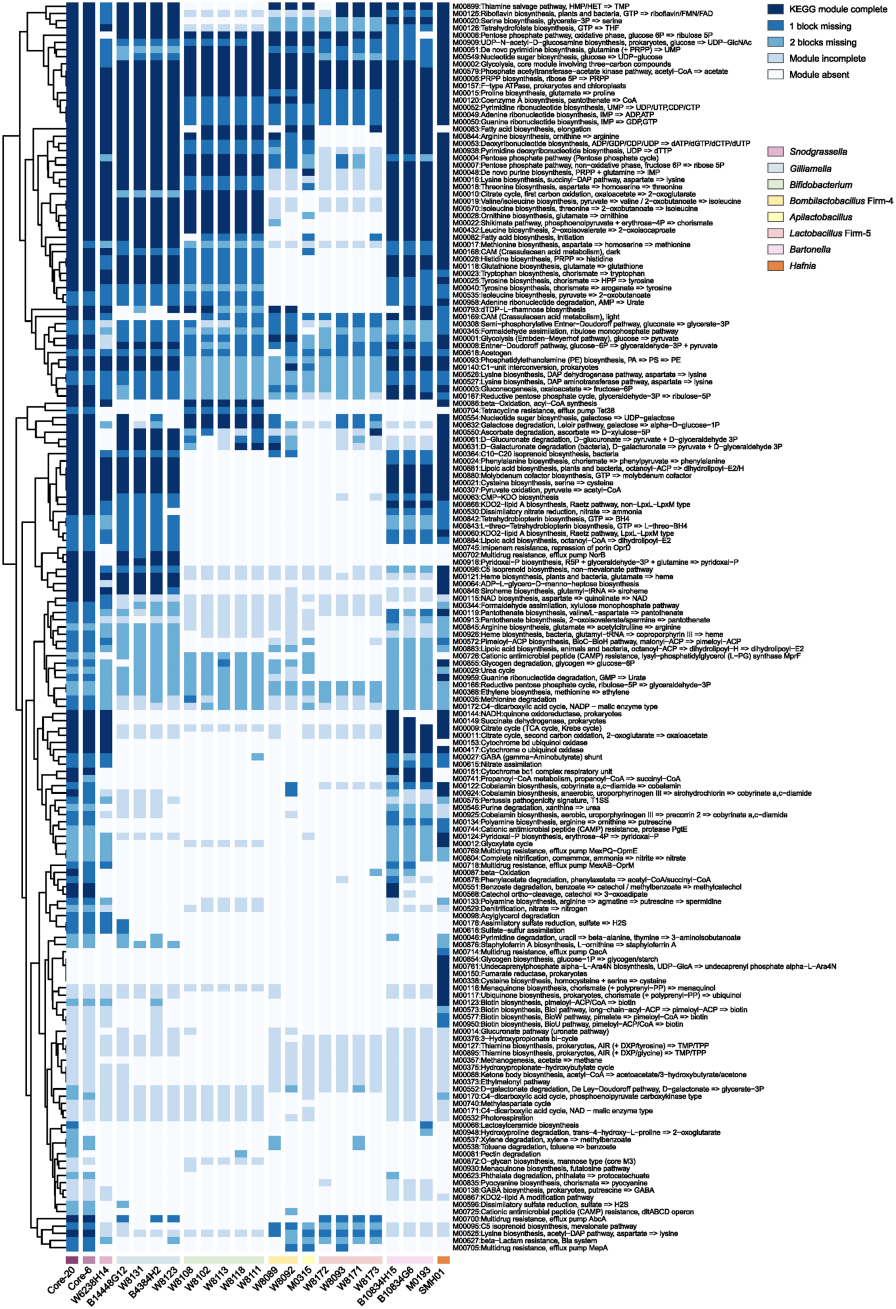
The presence and completeness of KEGG modules analysis of individual strains, the Core-6, and the Core-20 community. A hierarchical clustering heat map of KEGG module distribution in the draft genomes and artificial metagenomes. The comparison of the Core-6 and Core-20 shows functional similarity. We also found highly overlapping functions between *H. alvei* and the Core-20 community, indicating its fitness and potential virulence. The color code indicates the presence and completeness of each KEGG module, expressed as a value between module complete (dark blue) and module absent (white).

Protection against *H. alvei* by the Core-20 community supports immune regulation as a factor in pathogen defense. To gain insights into the potential functional capabilities of the Core-20 to activate immunologic responses, we sequenced and annotated the individual genomes of the 20 strains and mapped the predicted protein sequences against the KEGG database. Artificial metagenomes of the Core-6 and Core-20 were generated by merging the contigs of individual strains. The presence and completeness of KEGG modules were determined for individual genomes of 20 strains, the Core-6 and Core-20 (Figure 4). After hierarchical clustering, we observed different functional groups depending on their phylogenetic relatedness incidentally. The majority of strains share highly conserved modules, including phosphate acetyltransferase-acetate kinase pathway (M00579), PRPP biosynthesis (M00005), F-type ATPase (M00157), various carbohydrate metabolism pathways and multiple amino-acid and nucleoside biosynthesis pathways. Additionally, modules more prominent in *Gilliamella* strains comprised pyridoxal-p biosynthesis (M00916) and carbohydrate degradation modules, such as ascorbate, D-glucuronate, and D-galacturonate (M00550, M00061, M00631). We also found nucleotide sugar biosynthesis (M00554), galactose degradation (M00632), and beta-oxidation (M00086) modules enriched in *Bifidobacterium* strains. In total, the comparison of the Core-6 and Core-20 shows functional similarity. However, there were still several modules enriched in the Core-20, including cobalamin anaerobic biosynthesis (M00924), beta-oxidation (M00087), propanoyl-CoA metabolism (M00741), d-galactonate degradation(M00552), pectin degradation (M00081), and hydroxyproline degradation (M00948). Additionally, we also estimated the complement of KEGG modules for the genome of *H. alvei*, and we found highly overlapping functions with the Core-20 community, indicating its fitness and potential virulence. At the same time, several pathways were found enriched in *H. alvei*, such as glycogen biosynthesis (M00854), undecaprenylphosphate alpha−L − Ara4N biosynthesis (M00761), fumarate reductase (M00150), cysteine biosynthesis (M00338), menaquinone biosynthesis (M00116), ubiquinone biosynthesis (M00117), and multiple pathways of biotin biosynthesis. Overall, we speculated that receptors or products from carbohydrate, fatty acid, and amino acid metabolism could probably display a key role in regulating the immune system.

**Figure 5:**
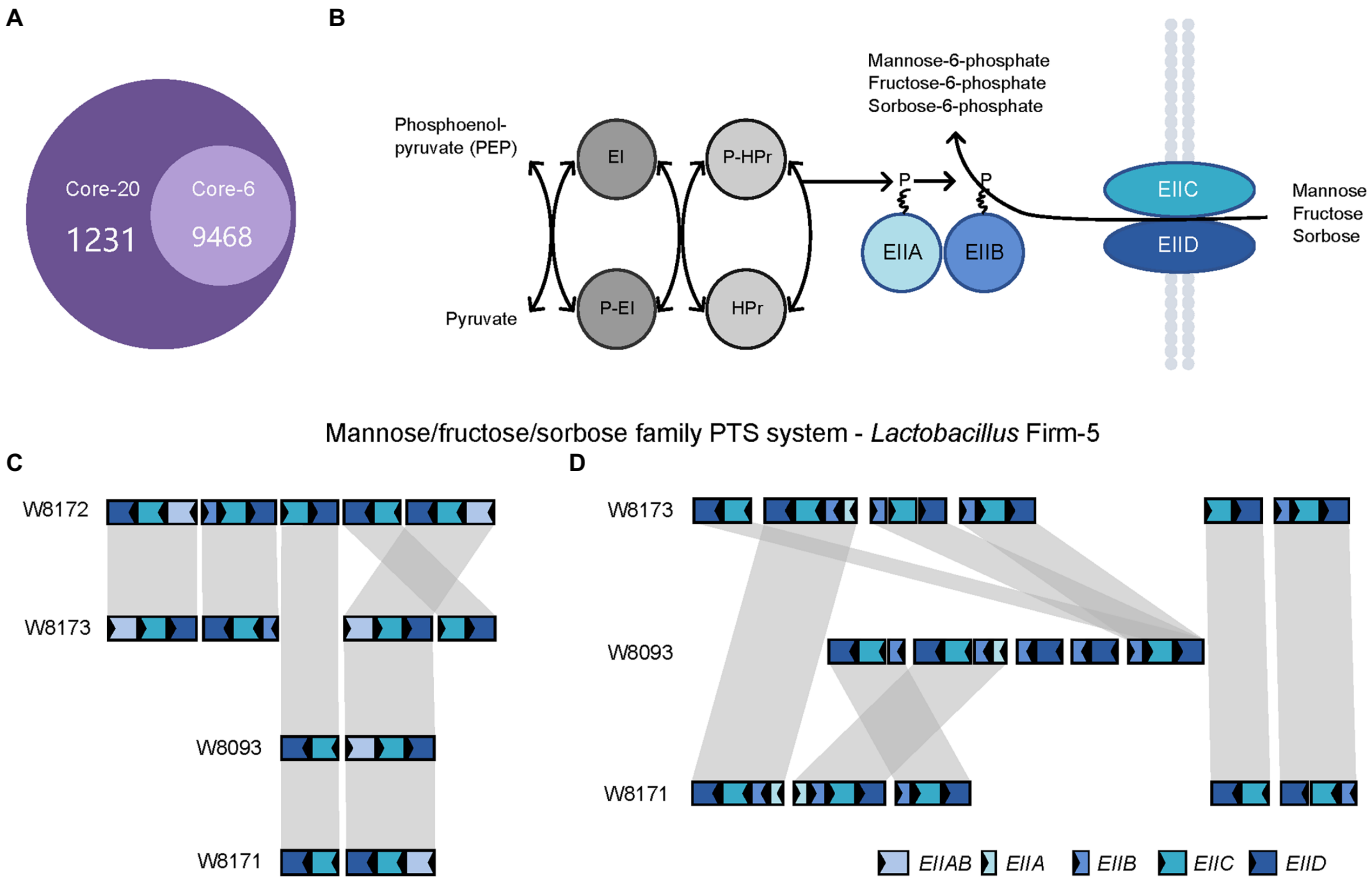
The PTS system enriched in the Core-20 community might trigger the immune response offering protection. **(A)** The Venn diagram was generated using OrthoVenn2. Results showed the number of shared orthologous clusters of protein-coding genes between artificial metagenomes. **(B)** Diagrammatic representation of the bacterial phosphotransferase signal transduction pathway (Mannose/fructose/sorbose family PTS system as an example). General phosphoryl and sugar transport reaction catalyzed by the PTS. Sugars are transported and concomitantly phosphorylated by the PTS. **(C,D)** In the genus *Lactobacillus* Firm-5, gene loci of mannose/fructose/sorbose family PTS system **(C)**, shared in 4 strains or **(D)**, unique to strains in the Core-20. Homologous genes are connected by gray bars. We found that W8173, W8093, and W8171, three stains specific to the Core-20, harbored their unique clusters of EIIA, EIIB, EIIC, and EIID, indicating that membrane-bound EII of phosphotransferase system could probably trigger an immune response.

Next, we investigated the genes specific to the Core-20 but not present in the Core-6, potentially associated with the capacity to trigger the immune system. The comparative analysis found that 3,206 genes unique to the Core-20 were enriched in 1,231 Gene Ontology clusters (Figure 5A). Notably, the enriched GO among all identified clusters was the phosphoenolpyruvate-dependent sugar phosphotransferase system (PTS, GO:0009401), a complex enzyme system functioning in the detection, transport, and phosphorylation of various sugar substrates (Kotrba et al., 2001; Gabor et al., 2011). The PTS is comprised of two general cytoplasmic components, enzyme I (EI) and histidine phosphoryl carrier protein (HPr), and membrane-bound sugar-specific multidomain enzymes II (EII). Each EII complex consists of one or two hydrophobic integral membrane domains (domains C and D) and two hydrophilic domains (domains A and B; Figure 5B). Mannose/fructose/sorbose family PTS system was observed, and four genes, including EIIAB, EIIB, EIIC, and EIID, were shared in four strains from the genus *Lactobacillus* Firm-5 (Figure 5C). Interestingly, W8173, W8093, and W8171, three stains specific to the Core-20, harbored their unique clusters of EIIA, EIIB, EIIC, and EIID (Figure 5D). Taken into account that both IIC and IID components of the mannose phosphotransferase system are involved in recognition of antimicrobial peptides (Kjos et al., 2010; Zhou et al., 2016), our results indicated that membrane-bound EII of phosphotransferase system could probably trigger an immune response, causing protection in the Core-20 bees.

### Stability transmission of Core-20 community during successive passaging *in vivo*

**Figure 6:**
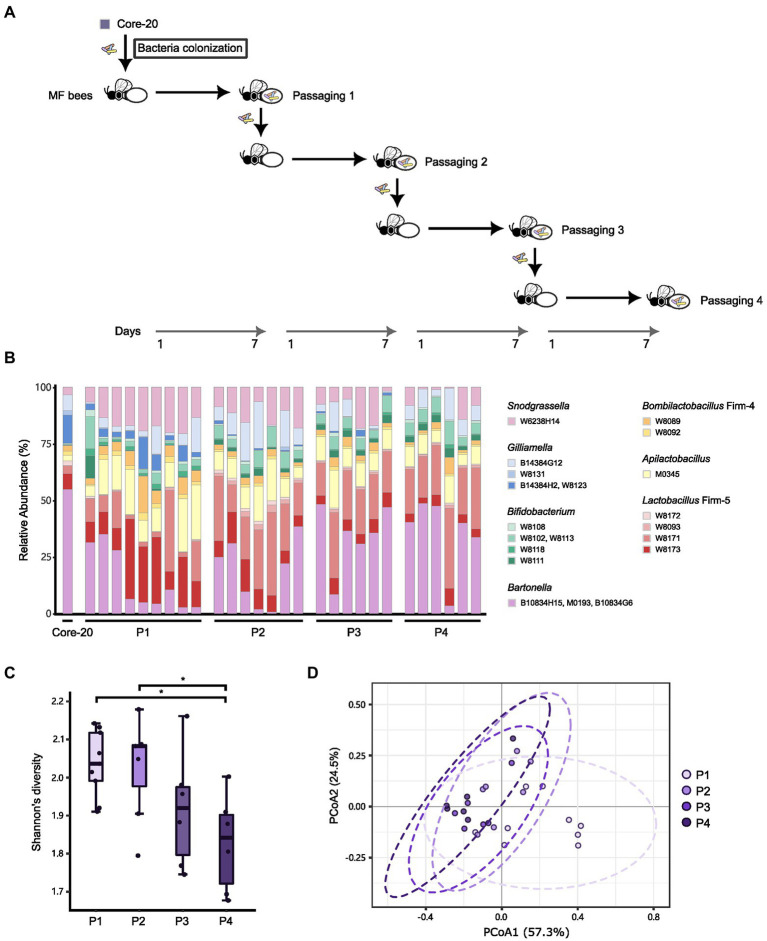
The Core-20 community stably colonized honeybees for four passages. **(A)** Experimental design for passaging transmission. **(B)** Relative abundance of strains in the Core-20 during four passages. All strains except *G.* sp. W8131 were detected individually in bee gut samples among passages. **(C)** Box plots of Shannon’s alpha diversity index at each passage. Results showed a slight decrease in alpha diversity. **(D)** Principal coordinates analysis (PCoA) plot of Bray–Curtis dissimilarity among samples. We found that the last three passages showed community similarity except for P1.

Due to the potential of the Core-20 to inhibit pathogens and shape the host immune system, we wonder whether the Core-20 community can stably colonize the honeybee gut over several passages. Microbiota-free bees were inoculated with the frozen mixtures of the Core-20 colony and sampled the whole gut on Day 7. The gut microbiota was mixed and passed on throughout four passages: passage 1 (P1), P2, P3, and P4. At the end of each passage, bacterial communities were sequenced by amplicon sequencing of the variable regions 3 and 4 of the 16S rRNA gene (Figures 6A,B). All strains except *G.* sp. W8131 were detected individually in bee gut samples among passages, indicating W8131 either is below the detection limit or does not colonize. The relative abundance of *Bifidobacterium*, *Snodgrassella*, and *Apilactobacillus* increased during passaging. Notably, *G. apicola* W14384G12 and *L. melliventris* W8171 were dominant within their genus, respectively. The relative abundance of *Bartonella* was maintained at a relatively stable level during transmission, suggesting the restriction and regulation of honeybee hosts to gut microbiota. We also found an overall decrease in alpha diversity over time across the four passages (Figure 6C, Tukey honest method, *p* < 0.05 for P1-P4, P2-P4) and a significant difference between P1 and the other passages in beta-diversity measured by Bray–Curtis dissimilarity (Figure 6D, PERMANOVA, *p* = 0.013 for P1-P2, *p* = 0.004 for P1-P3, *p* = 0.001 for P1-P4). Our findings indicated that strains of the Core-20 community display stable coexistence after slight fluctuations in species abundances and biomass during P1. In summary, these data suggest that the Core-20 community maintains stability despite fluctuations over the course of passage.

## Discussion

While early culture-based studies demonstrated that honeybee gut symbionts could be cultured *in vitro*, induce host immune response, and confer protection against pathogens after inoculation, little is known about the capacity of these isolates deposited in one colony. In this study, we assembled a defined microbial consortium of honeybees (the Core-20 community) based on the phylogeny analysis, which strongly inhibits *H. alvei*. Following exposure, *H. alvei* can grow to high loads (10^9^ CFU per gut), produce inflammatory reactions, and potentially result in host mortality. We focused on the expansion of *H. alvei* infection, which is primarily influenced by the gut microbiota, and carried out comprehensive investigations on the mechanism of colonization resistance by the gut microbiota. The Core-20 community could trigger upregulation of AMPs and precise *H. alvei* prevalence, indicating immune priming underlies part of the defined community protective effect. Functions related to carbohydrate utilization and the PTS system were represented in genomic analysis of the Core-20 community, which might play a role in immune system stimulation. Additionally, we found that the Core-20 community is able to colonize the honeybee gut over four passages stably. Our findings highlight a defined microbial community could offer protection *via* host–microbe interaction (for example, regulating the host immune system), suggesting that the Core-20 community could be used for gut microbiota research in honeybees.

A major function of the stable gut microbiota is to provide colonization resistance, preventing pathogens from colonizing and causing long-term infection and even mortality. Ghimire et al. identified *Clostridioides difficile*-inhibiting strains through single strain versus pathogen coculture assays *in vitro*. However, when they came to investigate how changes in the combinatorial assembly of bacteria might affect the inhibition capacity, their results demonstrated that new phenotypes masking the individual strain phenotype could emerge depending on the composition of the mix. For instance, bacterial consortia, where all the strains individually showed inhibition, display the enhancement of *C. difficile* growth (Ghimire et al., 2020). Moreover, germ-free mice colonized with members of the altered Schaedler flora (ASF), a bacterial consortium consisting of eight mouse-derived strains, provided insufficient colonization resistance to *Salmonella enterica* serovar Typhimuriumthe. However, enforced with three facultative anaerobes in Oligo-MM^12^ mice prevent infection completely (Brugiroux et al., 2016). Here, *B. choladocola* B10834H15 from *Bartonella* and *B. choladohabitans* W8113 from *Bifidobacterium* significantly inhibited the growth of *H. alvei*. In previous studies, *Bifidobacterium* of honeybees could produce antimicrobial substances *in vitro* to inhibit other microorganisms, contributing to the resistance of pathogenic bacteria for the host (Forsgren et al., 2010; Vásquez et al., 2012; Butler et al., 2013). In addition, *Bombella apis* has been evidenced to benefit the larval development of honey bees and protect larvae against fungal pathogens (Liu et al., 2022). Notably, the Core-6 community could increase the growth of *H. alvei*. In contrast, single strains and the Core-20 effectively inhibited *H. alvei* (Figure 2C), demonstrating that a defined bacterial community could offer the inhibition capacity as individual strains. The microbe-microbe interaction needs to be concerned with designing defined pathogen-inhibiting bacterial mixtures *in vivo*.

The mechanisms that regulate the ability of the microbiota to restrain pathogen growth are complex, including induction of host immune responses, localization to intestinal niches, and competitive metabolic interactions (Kamada et al., 2013). AMPs can maintain gut microbiota homeostasis by selectively inhibiting foreign bacteria and keeping native symbionts from over-proliferating (Kwong et al., 2017). The synthesis and secretion of AMPs is a highly regulated process, mainly controlled by the Toll and Imd pathways (Lourenço et al., 2013, 2018; Danihlík et al., 2015). Specific gut symbionts, such as *S. alvi*, *A. kunkeei*, *Frischella perrara*, and *L. apis*, have been confirmed to induce honeybee innate immune response. They upregulate the Toll and Imd pathway, leading to AMPs expression (Emery et al., 2017; Daisley et al., 2020b; Lang et al., 2022). Considering that the Core-6 consisted of microbes that were able to induce the immune response, the whole gut microbiota balance composition could be more important for regulating the immune system. The Core-20, a high-species-diversity colony, had more significant upregulation of the immune regulatory genes and AMPs genes encoding abaecin, apidaecin, hymenoptaecin, and defensin-1 (Figure 3), suggesting the ability of the Core-20 community in stimulating host innate immune system through their regulators and effectors.

Biofilm and the outer membrane protein, such as the S-layer protein unique to *L. apis* W8172, could be potential drivers of the host immune response. We used KEGG modules to character gene sets linked to specific metabolic capacities and OrthoVeen2 to compare and annotate orthologous gene clusters among multiple genomes (Figures 4, 5). Results showed that the PTS system was significantly enriched in the Core-20 community. The PTS system is a highly conserved phosphotransfer cascade whose components modulate many cellular functions in response to carbohydrate availability (Houot et al., 2010). Previous studies have elucidated the importance of bacterial PTS system for honeybees, including detoxifying specific nectar components (Engel and Moran, 2013a), nutrient metabolic transformations (Lee et al., 2015), and adaptation to the diet and gut environment of the honeybee. PTS system of *Enterococcus faecalis* could increase proinflammatory cytokine secretion by colon tissue and macrophages to enhance colonization in mice (Fan et al., 2019). Besides, the PTS system of *Vibrio cholerae* display control of carbohydrate transport and activation of biofilm formation on abiotic surfaces (Houot et al., 2010). Additionally, EIIC and EIID from the mannose/fructose/sorbose family PTS system, the membrane-banding proteins, is responsible for specific targeting by antimicrobial peptides, indicating their potential to regulate the immune system (Diep et al., 2007; Kjos et al., 2010; Zhou et al., 2016).

According to Rolf Freter’s nutrient niche theory, a pathogen can only invade if it is able to use a specific limiting nutrient more efficiently than the rest of the community, which means colonization resistance against pathogens is affected by efficient restriction of all available nutrient niches by a complex microbial community (Freter et al., 1983). Invasion theory Figures out that biotic selection could be the critical determinant (Dillon et al., 2005; van Elsas et al., 2012; Mallon et al., 2015; Ketola et al., 2017). Higher diversity communities can competitively exclude an invader by reducing the availability of ecological niches and efficiently utilizing resources (Hromada et al., 2021). Thus, the protective effect is probably provided through antagonism between microbes (Chiu et al., 2017; Ubeda et al., 2017). In the case of an animal pathogen, three facultative anaerobes potentially prevent infection in Oligo-MM^12^ mice by filling up the niche space that is preferred by *S.* Tm (Brugiroux et al., 2016). Previous studies showed that *H. alvei* reduced nitrates and fermented l-arabinose, glycerol, maltose, d-mannitol, d-mannose, l-rhamnose, trehalose, and d-xylose (Møller, 1954; Janda et al., 2005; Tian and Moran, 2016; Erban et al., 2017). Genomic analysis reveals that *H. alvei* harbors various carbohydrate degradation modules and has similar functions as the Core-20 (Figure 4), suggesting its ability to grow in the honeybee gut and compete for multiple carbohydrates. *Gilliamella*, a primary polysaccharide degrader in the honeybee gut, utilizes mannose, arabinose, xylose, or rhamnose (monosaccharides that can cause toxicity in bees; Zheng et al., 2016, 2017, 2019). Functions for carbohydrate use and PTS systems are represented in genomic analysis of the Core-20 community, which may also promote colonization resistance by competition for limited nutrients that *H. alvei* presumably depends on. Our findings implied that protection by the *Gilliamella* and the Core-20 bees occurs *via* occupation of niche space (for example, consumption of carbohydrates) that can no longer be exploited by *H. alvei*. Loss of microbial diversity might create ecological niches that pathogens can use, underlying why bees colonized with low-complexity gut microbiota, such as the Core-6, are more susceptible to *H. alvei* infection. Whereas, because the Core-20 had 14 strains more than the Core-6, it is conceivable that the Core-20 community could actually fill up the niche space that is preferred by *H. alvei* and thereby prevent infection.

The honey bee gut microbiota is dominated by limited numbers of bacterial phylotypes, commonly with species from the *Gilliamella*, *Snodgrassella*, *Lactobacillus* Firm-5, *Bombilactobacillus* Firm-4, *Bifidobacterium*, and *Bartonella* genera. Gut microbial communities influence host health in many ways, including food digestion, defense against pathogens, and modulation of behavior, development, and immunity (Engel and Moran, 2013a,b). Therefore, dysbiosis (microbial imbalance) may impact honeybee health and susceptibility to disease. Honeybees treated with tetracycline severely altered both the size and composition of the gut microbiome, decreasing the survival rate of bees and increasing susceptibility to opportunistic pathogens (Raymann et al., 2017; Lang et al., 2022). Here, the Core-20 consisted of typical isolates representing species in honeybee gut microbiota, which demonstrated transmission stability and functional redundancy during passages. Potentially, consequences of dysbiosis, such as nutritional impacts or heightened susceptibility to toxins, could be reduced through the development of alternative treatment methods, for example, adding the Core-20 to the bee hive.

In conclusion, we have assembled a minimal community of 20 bacterial strains that provided colonization resistance against *H. alvei*, elucidating the underlying molecular and functional mechanisms. The native gut symbionts are essential in the resistance to pathogen invasion. Such strain collections can yield insights into host-microbiota interactions, hoping to offer solutions to protect honeybees from pathogen infection.

## Data availability statement

The datasets presented in this study can be found in online repositories. The names of the repository/repositories and accession number(s) can be found in the article/Supplementary material.

## Author contributions

JW and HZ designed the research. JW, HL, and WZ collected the samples. JW, HL, and WZ performed the experiments and analyzed the data with contributions from YZ, LZ, HL, and YL. JW and HZ wrote the manuscript. All authors contributed to the article and approved the submitted version.

## Funding

This work was supported by the National Key R&D Program of China (grant no. 2019YFA0906500).

## Conflict of interest

The authors declare that the research was conducted in the absence of any commercial or financial relationships that could be construed as a potential conflict of interest.

## Publisher’s note

All claims expressed in this article are solely those of the authors and do not necessarily represent those of their affiliated organizations, or those of the publisher, the editors and the reviewers. Any product that may be evaluated in this article, or claim that may be made by its manufacturer, is not guaranteed or endorsed by the publisher.

## Footnotes

^1^ https://www.genome.jp/kegg/kaas/

^2^ https://www.genome.jp/kegg/mapper/

^3^ http://orthovenn2.bioinfotoolkits.net

## Supplementary material

SUPPLEMENTARY TABLE S1 qPCR primer sequences for gene expression and bacterial load.

SUPPLEMENTARY FIGURE S1 Differences of the V3–V4 region of 16S rRNA genes of strains in the relevant genus. Our results indicated the feasibility of using 16S rRNA genes V3–4 region for species classification.
