# Supplementary material for: Stably transmitted defined microbial community in honeybees preserves Hafnia alvei inhibition by regulating the immune system
Source: Front Microbiol. 2022 Dec 1;13:1074153. doi: 10.3389/fmicb.2022.1074153 (PMC9751035; doi:10.3389/fmicb.2022.1074153)
Supplement: SUPPLEMENTARY TABLE S1 — qPCR primer sequences for gene expression and bacterial load. [file Image_1.pdf]

Supplementary Information

Table S1. qPCR primer sequences for gene expression and bacterial load

Gene	Forward	Reverse	Reference
Toll	TAGAGTGGCGCATTGTCAAG	ATCGCAATTTGTCCCAAAAC	(Lang et al., 2022)
Spz4	CAACGAATTCAGGGACGAGG	AGTAGTGCCGGGGAAATTCA	This study
Cactus-1	CTATCGTGGAGAAACTGCGTAT	TCAGGAAGTGGTTCTGGTATTG	(Lang et al., 2022)
Cactus-2	ATCAGACGGCTCTGCTCTAT	TCGTCTTCGTCAGTGGTATCT	(Lang et al., 2022)
Dorsal	AGAGATGGAACGCAGGAAAC	TGACAGGATATAGGACGAGGTAA	(Lang et al., 2022)
PGRP-LC	TCCGTCAGCCGTAGTTTTTC	CGTTTGTGCAAATCGAACAT	(Lang et al., 2022)
Dredd	GCGTCATAAAGAAAAAGGATCA	TTTCGGGTAATTGAGCAACG	(Lang et al., 2022)
Relish	GGAGCTGATCCAAATCGAAC	AGTGGCATCCATCCATCATT	(Lang et al., 2022)
Abaecin	TCGGATTGAATGGTCCCTGAC	ATCTTCGCACTACTCGCCAC	(Lang et al., 2022)
Apidaecin	GTAGGTCGAGTAGGCGGATCT	TTTTGCCTTAGCAATTCTTGTTG	(Lang et al., 2022)
Hymenoptaecin	GTCGTCCATCCTTGGACATT	TTTCCCAAACTCGAATCCTG	(Lang et al., 2022)
Defensin-1	TGCGCTGCTAACTGTCTCAG	AATGGCACTTAACCGAAACG	(Lang et al., 2022)
Defensin-2	GCAACTACCGCCTTTACGTC	GGGTAACGTGCGACGTTTTA	(Lang et al., 2022)
Lysozyme	ACACGGTTGGTCACTGGTCC	GTCCCACGCTTTGAATCCCT	(Lang et al., 2022)
Hafnia	CGAGGAGGAAGGCATTGTGG	CCCAAGTTAAGCTCGGGGAT	(Lang et al., 2022)
Actin	TGCCAACACTGTCCTTTCTG	AGAATTGACCCACCAATCCA	(Lang et al., 2022)

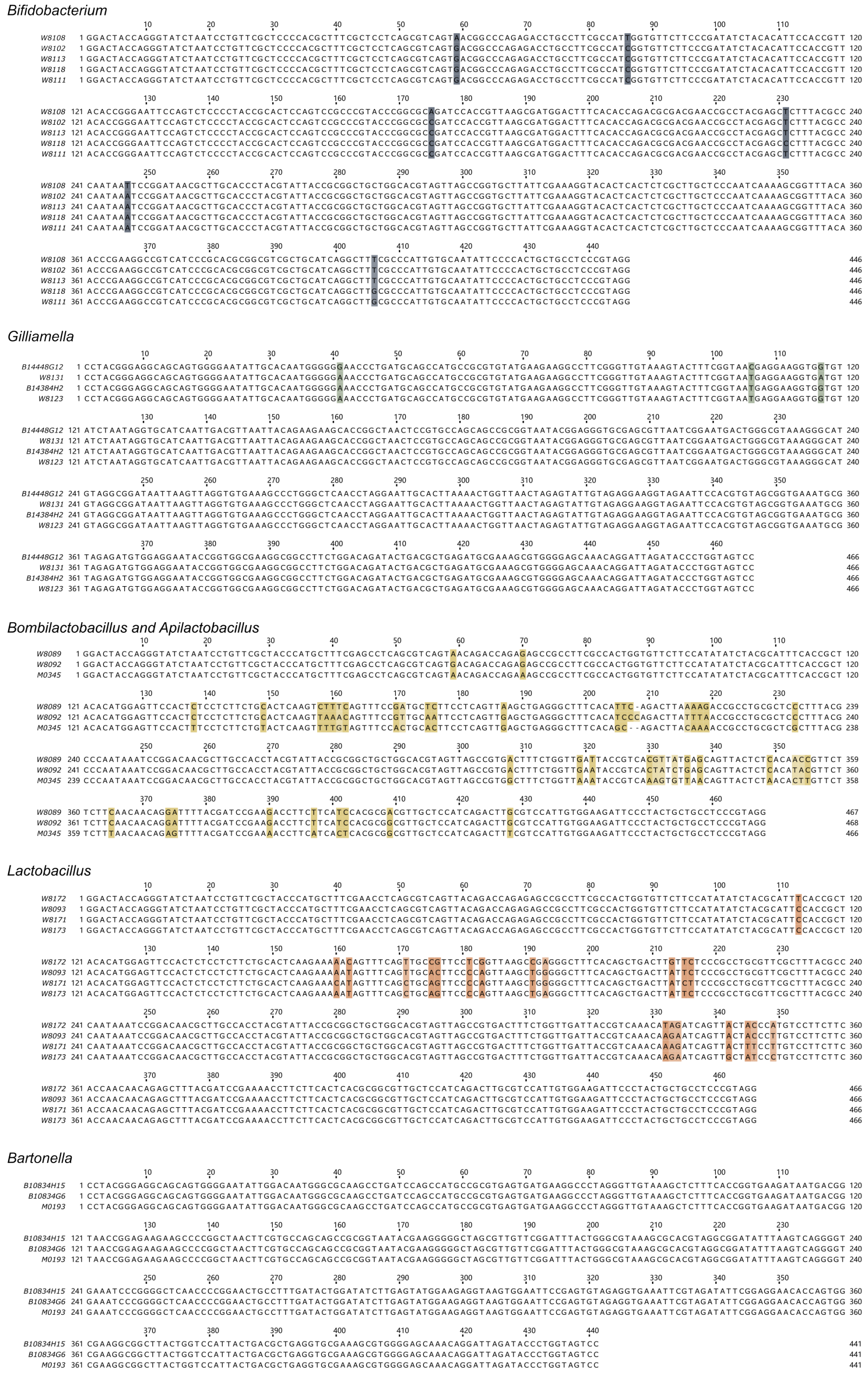

Fig. S1. Differences of the V3 - V4 region of 16S rRNA genes of strains in the relevant genus. Our results indicated the feasibility of using 16S rRNA genes V3 - 4 region for species classification.
